# Drug–drug interactions between propofol and ART drugs: Inhibiting neuronal activity by affecting glucose metabolism

**DOI:** 10.1111/cns.14437

**Published:** 2023-08-31

**Authors:** Sijun Li, Yanqing Zheng, Qian Long, Jianhong Nong, Honghua Shao, Gang Liang, Fengyao Wu

**Affiliations:** ^1^ Department of Internal Medicine The Fourth People's Hospital of Nanning Nanning China; ^2^ Infectious Disease Laboratory The Fourth People's Hospital of Nanning Nanning China; ^3^ Department of Clinical Laboratory The Fourth People's Hospital of Nanning Nanning China; ^4^ Department of Anesthesiology The Fourth People's Hospital of Nanning Nanning China

**Keywords:** ART drugs, glucose, glucose transporter 3, neuron, propofol

## Abstract

**Background:**

The use of two or more drugs carries the potential risk of drug–drug interactions (DDIs), which may result in adverse reactions. Some human immunodeficiency virus (HIV)‐infected patients who receive antiretroviral therapy (ART) may require general anesthesia with propofol (PRL) before undergoing surgical treatment. Both PRL and ART drugs may lead to neuronal dysfunction, which can be accompanied by energy metabolism disorders. Neurons take in glucose mainly through glucose transporter 3 (Glut3) which is specifically expressed on the cell membranes of neurons. However, to date, no study has examined whether the DDIs of PRL and ART drugs interfere with glucose metabolism and Glut3 expression in neurons.

**Methods:**

An in vitro model was constructed using the primary cultures of neurons. PRL and ART drugs (EFV, AZT, and 3TC), were added at different concentrations (low, medium, and high). The neurons were exposed to the drugs for 1, 4, 8, and 12 h. The optimal drug concentration and exposure time were selected. The cellular survival rate, glucose concentration, electrophysiology, and the expression of Glut3 were detected.

**Results:**

There were no significant changes in the cellular survival rates of the neurons that were exposed to both PRL and ART drugs at low concentrations for 1 h. However, the survival rates of the neurons decreased significantly as the drug concentrations and durations increased. The glucose concentration of the neurons that were exposed to both PRL and the ART drugs was significantly decreased. The glucose concentration of the neurons was not affected by any individual drug. The amplitude of the action potential and the expression of Glut3 were decreased in the neurons that were exposed to both PRL and ART drugs.

**Conclusions:**

The main adverse reactions induced by the DDIs between PRL and the ART drugs were decreased glucose metabolism and neuronal damage, which were caused by inhibiting the expression of Glut3. More importantly, we found that decreases in glucose metabolism predated neuronal damage.

## Graphical abstract

The main adverse reactions induced by the drug–drug interactions between propofol (PRL) and the antiretroviral therapy (ART) drugs were decreased glucose metabolism and neuronal damage, which were caused by inhibiting the expression of glucose transporter 3.

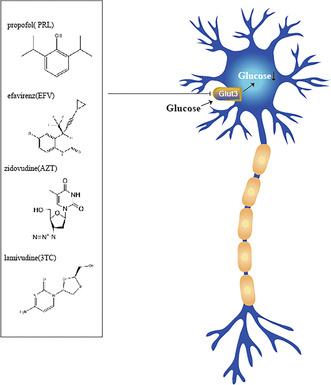

## 1 INTRODUCTION

The use of two or more drugs carries the potential risk of drug–drug interactions (DDIs), which may result in adverse reactions.^1^, ^2^ If there were sufficient data to support the use of common drugs in combination, such as antihypertensive drugs, anticancer drugs,^3^ and antituberculosis drugs,^4^ clinicians could anticipate and prepare for the adverse effects of DDIs.^5^, ^6^ Patients infected with human immunodeficiency virus (HIV) are often treated with combinations of multiple drugs. Due to the complexity of this disease and the immune dysfunction of these patients, unpredictable adverse reactions often occur.^7^ Thus, close attention needs to be paid to the adverse reactions induced by DDIs in the process of treating these patients with medication.

About 2.5 million people are newly infected with HIV each year, and about 2.1 million die from anti‐immune deficiency syndrome (AIDS)‐related illnesses each year.^8^ At the end of the last century, a new kind of treatment named anti‐retroviral therapy (ART) was introduced into clinical practice. ART has decreased mortality rates and the incidence of systemic opportunistic infections in AIDS patients by suppressing the systemic HIV viral load.^9^ Despite the advances brought about by ART, ART drugs have neurotoxic side effects that can cause central nervous system (CNS) dysfunction and lead to cognitive dysfunction.^10^ Funes HA et al.^11^ suggested that ART durgs alter mitochondrial respiratory function in neurons.

Approximately 20%–25% of HIV patients require surgical treatment.^12^ Propofol (PRL) is the most commonly used drug for general anesthesia. Long‐term clinical experience and experimental data have shown that PRL is harmless to most patients in reasonable doses.^13^ However, previous research has indicated that PRL can cause neurotransmitter metabolism disorders and even CNS damage, ultimately affecting the cognitive function of patients.^14^ Andrada et al.^15^ suggested that PRL has been shown to inhibit neuronal activity by enhancing the inhibitory function of neurons. Currently, it is not clear whether the DDIs caused by PRL combined with ART drugs affect the CNS.

The most important functional cells of the CNS are neurons, which are non‐renewable.^16^ The non‐renewable neuron is also an important factor in irreversible neurological impairment. In the process of CNS damage and disease development, neuronal dysfunction is accompanied by energy metabolism disorders.^17^ As long as these metabolic disorders are corrected in time, neurons can be effectively protected.^18^, ^19^ Neuron energy metabolism relies predominantly on glucose and oxygen utilization to generate biochemical energy in the form of adenosine triphosphate (ATP).^20^ Neurons take in glucose mainly through glucose transporter 3 (Glut3) which is specifically expressed on the cell membranes of neurons.^21^ The glucose is used as the original tricarboxylic acid cycle to produce ATP.^22^, ^23^ Glut3 belongs to the SoLute Carrier (SLC) family and is encoded by the SLC2A3 gene.^24^ A previous study showed that decreased Glut3 expression in neurons led to decreased glucose uptake efficiency, which in turn led to decreased cell activity in a model of cognitive dysfunction.^25^ The overexpression of Glut3 can effectively improve the cellular activity of neurons and cognitive function.^26^ Thus, Glut3‐mediated neuronal glucose metabolism is closely related to neuronal activity. However, to date, no study has examined whether the DDIs of PRL and ART drugs interfere with glucose metabolism and Glut3 expression in neurons.

To investigate whether PRL combined with ART drugs affected the glucose metabolism, cell activity, and Glut3 expression of neurons, we detected the survival rate, glucose concentration, electrophysiology, and Glut3 expression of primary neurons that were exposed to both PRL and ART drugs.

## 2 METHODS

### 2.1 Primary neurons exposed to drugs

A protocol was prepared before the study without registration. Primary cultured neurons were obtained from 12 newborn (24‐h‐old, 6–7 g) Sprague–Dawley rats. All the animals were obtained from the Animal Laboratory Center of Guangxi Medical University. The animal study was carried out at Guangxi Medical University. The animal experiments were performed under a project license (no. 202101023) granted by The Guangxi Medical University's Ethics Board and conducted in compliance with national guidelines for the care and use of animals. The minimum sample size for the experimental animals was calculated using statistical methods based on preexperimental data to reduce the number of animals and ensue the comparability of the experimental data as much as possible.

The primary neuron culture was carried out according to the method of Li et al.^27^ When the neurons had been cultured for 7 days, the neurons were identified and exposed to the drugs. The neurons in different wells were randomly grouped. No drugs were added to the control (Ctrl) group. The following drugs were used: PRL (20, 40, 80 mM), efavirenz (EFV) (4, 8, 16 mM), zidovudine (AZT) (4, 8, 16 mM), and lamivudine (3TC) (4, 8, 16 mM). A mixture of the four drugs was added to the medium in high, medium, and low concentrations. Based on their exposure to the drugs, the neurons were divided into the following three groups: (I) the low PRL + ART concentration group (the LPA group); (II) the medium PRL + ART concentration group (the MPA group); and (III) the high PRL + ART concentration group (the HPA group). The neurons were exposed to the drugs for 1, 4, 8, and 12 h.

### 2.2 3‐(4,5‐dimethylthiazol‐2‐yl)‐2,5‐diphenyltetrazolium bromide (MTT) analysis of neuronal survival rate

An MTT kit (Boster, AR1156) was used to detect the cell survival rates. MTT staining solution (10 μL) was added to each well, and the cells were cultured at 37°C for 4 h. Next, each Formanzan solution (100 μL) was added to each well, and the cells continued to be incubated at 37°C for 4 h. Absorbance was measured at 570 nm using an enzyme‐labeled instrument.

### 2.3 Glucose concentration measurement

The glucose concentration of the samples was detected using a glucose determination kit (Nanjing Jiengcheng, A154‐1‐1). The absorbance value of each sample was detected using an enzymic label at a wavelength of 505 nm. The following formula was used: Glucose content (mol/g protein) = [measured optical density (OD) value − blank OD value]/(standard OD value − blank OD value) × standard concentration/sample protein concentration (g/L).

### 2.4 Detection of the electrophysiology of the neurons

We used Digidata 1550B patch‐clamp amplifier to obtain electrophysiology of neurons. The neuronal action potentials were performed according to the method of Li et al.^27^

Miniature inhibitory postsynaptic currents (mIPSCs), mainly produced by postsynaptic gamma‐aminobutyric acid (GABA) type A receptors (GABAaRs),^28^ were recorded according to the method of Wyrembek et al.^29^ All the measurements were recorded at 25°C. The data were analyzed by Clampfit 10.7 and MiniAnal. The data of neuronal AP and mIPSCs were analyzed by Axon Digidata 1550B 16‐bit data acquisition system and pClamp 10.7 data acquisition software. In the in vitro model, a single neuron was considered an experimental unit. In each group, five neurons were selected for patch clamp detection.

### 2.5 Western blot

We extracted the total protein of neurons with Protein Extraction Kit (Invent Biotechnologies, SD‐001) and evaluated the protein concentrations with bicinchoninic acid assay kit (Beyotime, P0012S).^30^ We used sodium dodecyl‐sulfate polyacrylamide gel electrophoresis to separate the protein samples. Next, the protein was transferred to the nitrocellulose (NC) membrane (Merck Millipore Ltd) by wet transfers (Bio‐Rad). The blocked NC membrane was incubated with the primary antibodies at 4°C overnight. The primary antibodies were an anti‐Glut3 antibody (1:8000, Abcam, ab41525) and anti‐glyceraldehyde 3‐phosphate dehydrogenase (GAPDH) antibody (1:10,000, Sangon Biotech, D110016). Subsequently, the NC membrane was incubated with a secondary antibody (1:8000, Proteintech, SA00001‐2) for 1 h at room temperature. The immunoreactive bands were incubated with Chemiluminescent solution (EpiZyme, SQ201) and visualized by ChemiScope6000 system.^31^ ImageJ software was employed to quantify the band intensities of the western blot images. The protein levels of Glut3 were determined by the gray level of Glut3 normalizing to the gray level of GAPDH. Each experiment was repeated three times. Secondary antibody.

### 2.6 Immunofluorescence(IF) analysis

The neurons were fixed with 4% paraformaldehyde for 30 min, penetrated with 0.1% TritonX‐100 for 10 min, and blocked with 5% Bovine serum albumin (BSA) for 1 h. The neurons were stained using a multiplex fluorescent immunohistochemistry kit (absin, abs50012).^32^ The primary antibodies used included antineuron‐specific enolase (anti‐NSE) antibody (1:50, BOSTER, BM4495) and anti‐Glut3 antibody (1:100, Abcam, ab41525). An Olympus BX53 fluorescence mirror was employed to reveal the fluorescence signals of NSE (green) and Glut3 (red). NSE is a neuron‐specific marker that can be used to identify neurons. The ImageJ plug‐in was used to calculate the purity of the neurons and the average optical density (AOD) of Glut3.

### 2.7 Statistical analysis

All the experiments were repeated three times. The data describe the biological replicates. The data are expressed as the mean ± standard deviation. An independent sample *t* test was used to assess the significance of the differences between the 2 data groups. An analysis of variance was used to assess the significance of the differences between >2 types of data groups. SPSS 25.0 was used for the statistical analysis. A *p* value of <0.05 was considered statistically significant.

## 3 RESULTS

### 3.1 The cell survival rate and glucose metabolism of neurons

**FIGURE 1:**
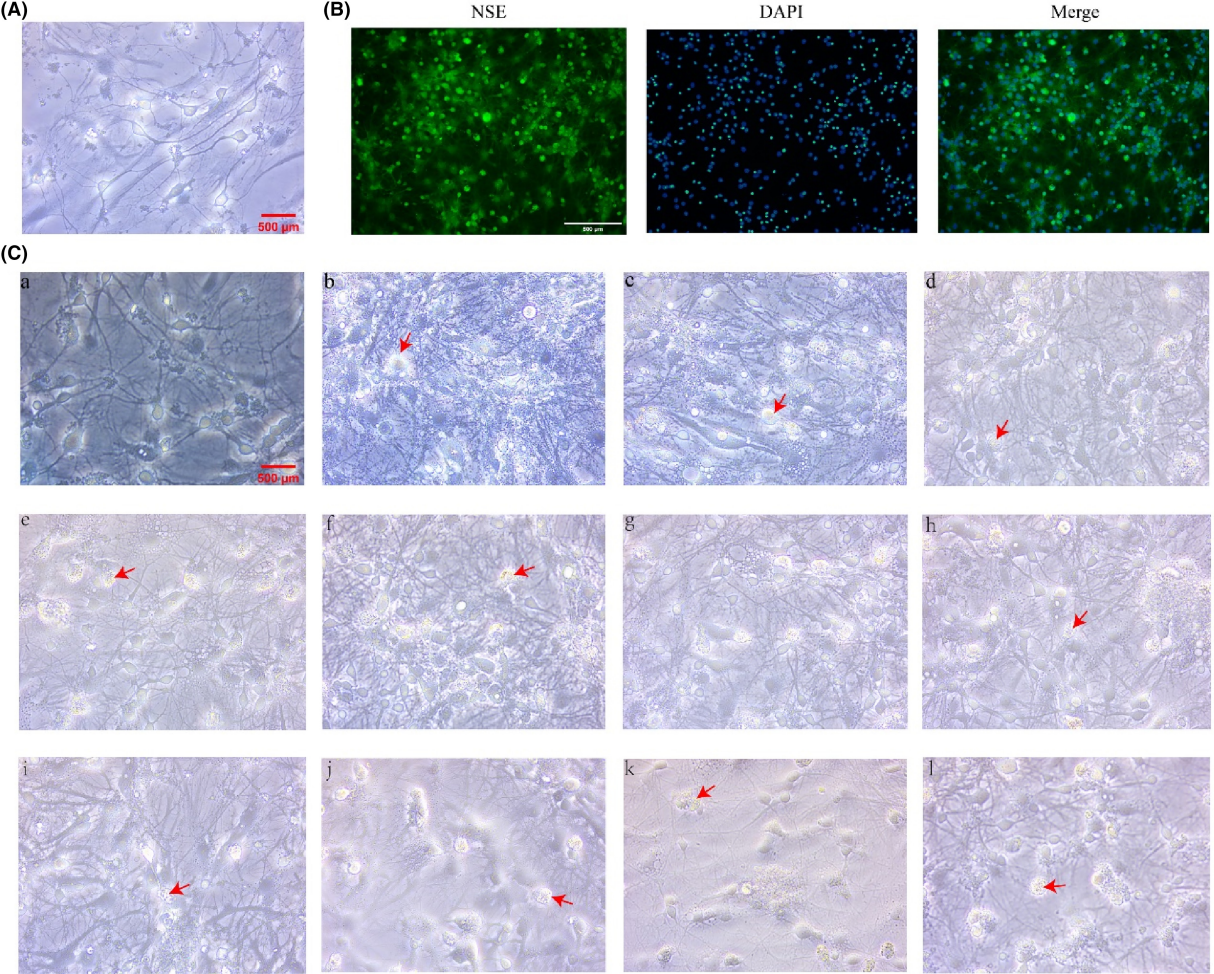
Neuron morphology. (A) The neurons in the Ctrl group (×400): the neuronal bodies were full, and the axons were clear. Scale: 500 μm. (B) The purity of the neurons (immunofluorescence) (×100): the NSE staining of neurons showed green fluorescence, and cell purity accounted for >90%. Scale: 500 μm. (C) The effects of PRL and the ART drugs on neuronal morphology (×400): (a) LPA‐1‐h: the neuronal bodies were full, and the axons were clear in the LPA‐1‐h group, and there was no significant difference compared with the neurons in the Ctrl group; (b–l) the neurons were swollen and ruptured in the other groups (b, LPA‐4‐h; c, LPA‐8‐h; d, LPA‐12‐h; e, MLA‐10‐h; f, MLA‐4‐h; g, MLA‐8‐h; e, MLA‐12‐h; f, HLA‐1‐h; g, HLA‐4‐h; h, HLA‐8‐h; l, HLA‐12‐h). Scale: 500 μm. Red arrows: swollen and ruptured neurons. ART, antiretroviral therapy; Ctrl, control; HLA, high PRL + ART concentration; LPA, low PRL + ART concentration; MLA, medium PRL + ART concentration; NSE, neuron‐specific enolase; PRL, propofol.

**FIGURE 2:**
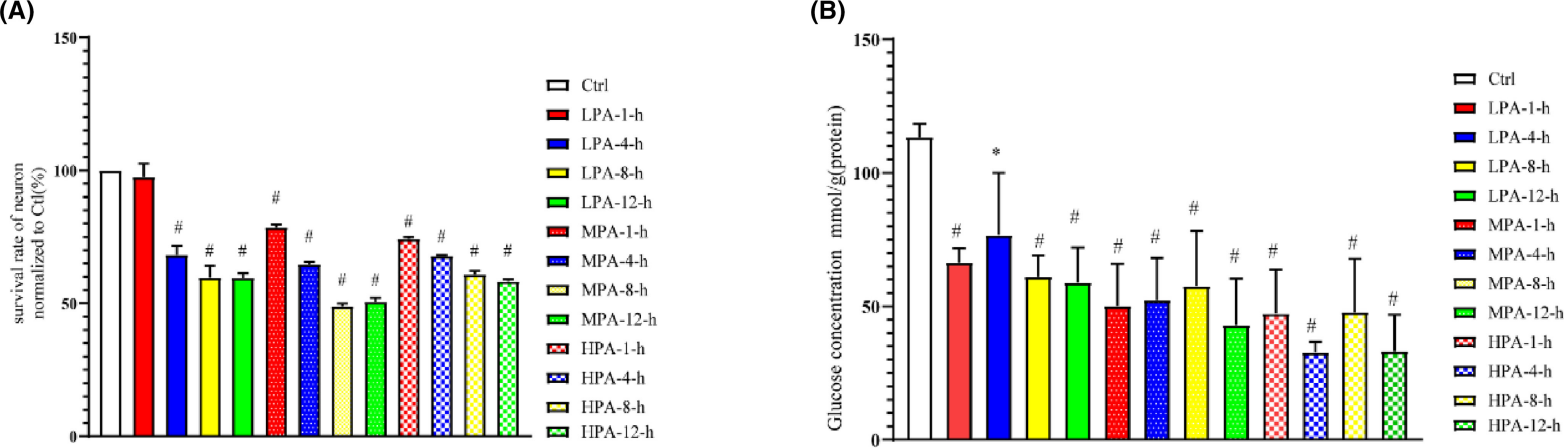
Survival rate, glucose concentration, and expression of Glut3 in the neurons. (A) The survival rates of the neurons: the survival rate of the neurons was the highest in the LPA‐1‐h group, and the other groups showed a significant decrease. (B) The effect of PRL + ART on the glucose concentration of the neurons: the glucose concentration of the neurons was decreased. *n* = 5; *p < 0.05, ^#^ *p* < 0.01 versus Ctrl. ART, antiretroviral therapy; Ctrl, control; Glut3, glucose transporter 3; HPA, high PRL + ART concentration; LPA, low PRL + ART concentration; MPA, medium PRL + ART concentration; PRL, propofol.

The bodies of neuron were full, and the synapses were obvious (Figure 1A). The number of green cells that were labeled by the NSE was counted, and the purity of the neurons reached >90% (Figure 1B). After exposure to PRL and the ART drugs, the cells of the LPA‐1‐h group were full, the neural network was clear, and there was no significant difference between the LPA‐1‐h group and the Ctrl group (Figure 1C). However, the neurons were swollen and ruptured in the other groups. We found that the survival rate of the neurons in the LPA‐1‐h group was similar to that in the Ctrl group, while the survival rate of the neurons in the other groups was significantly decreased (vs. Ctrl, *p* < 0.01, Figure 2A). The glucose concentration results showed that the glucose concentration was decreased in the neurons exposed to PRL and ART drugs at different time periods (vs. Ctrl, *p* < 0.01, Figure 2B). We noted that the level of glucose decreased without any significant change in the survival rate of the neurons in the LPA‐1‐h group, which met our subsequent experimental requirements.

### 3.2 Neuron activity

**FIGURE 3:**
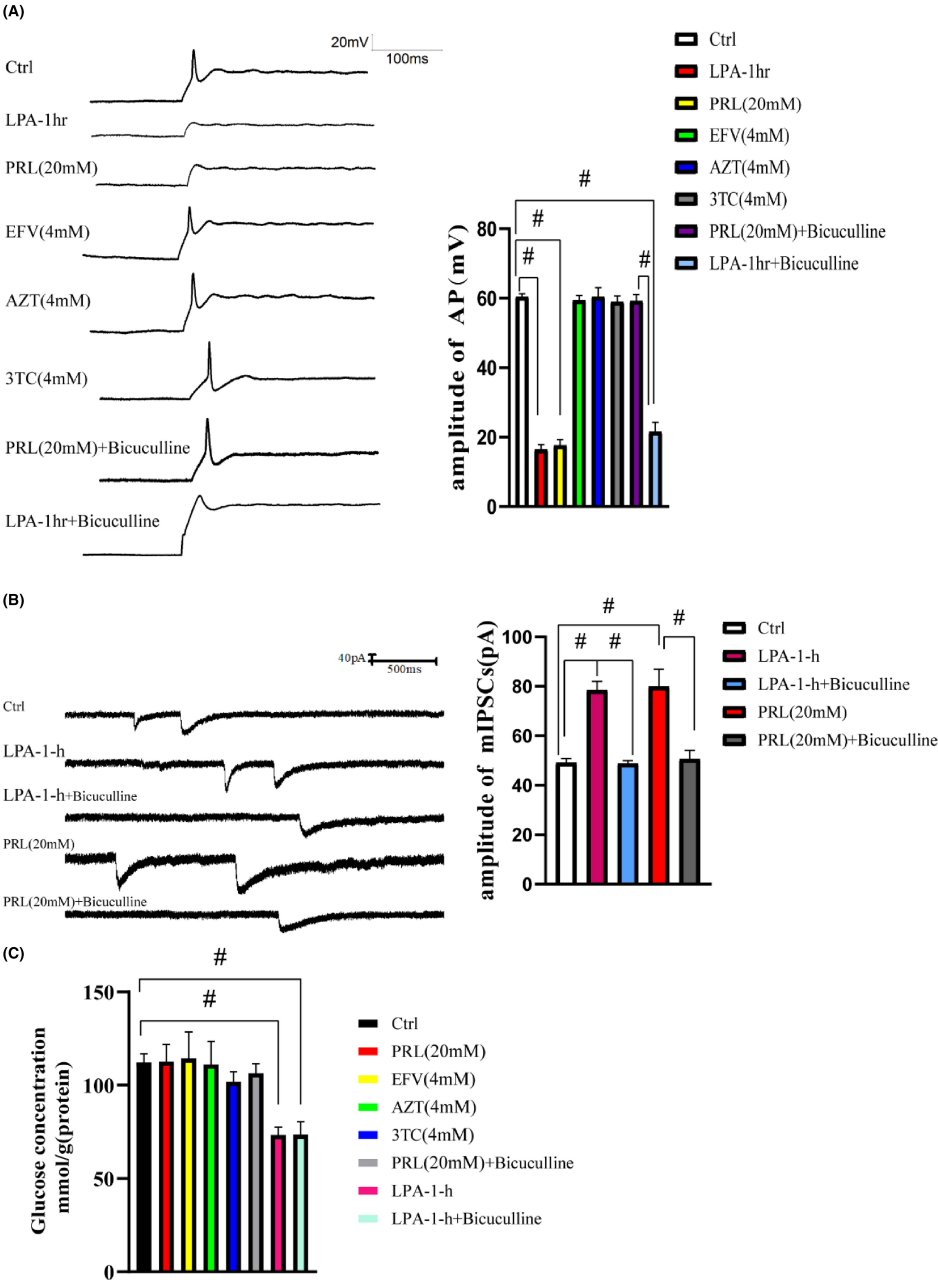
Neuronal activity. (A) The AP of the neurons: the AP amplitude was decreased in the neurons of the LPA‐1‐h group; the AP amplitude was decreased in the neurons of the PRL group; the AP amplitude was decreased in the neurons of the LPA‐1‐h + Bicuculline group. (B) The mIPSCs of the neurons: The mIPSC amplitude was increased in the neurons of the LPA‐1‐h group; the mIPSC amplitude was increased in the neurons of the LPA‐1‐h + Bicuculline group; the mIPSC amplitude was increased in the neurons of the RPL group; the mIPSC amplitude was increased in the neurons of the PRL + Bicuculline group. (C) The effect of each individual drug on the glucose concentration of the neurons: the glucose concentration of the neurons did not change significantly. *n* = 5, ^#^ *p* < 0.01. 3TC, lamivudine; AP, action potential; ART, antiretroviral therapy; AZT, zidovudine; Ctrl, control; EFV, efavirenz; LPA, low PRL + ART concentration; mIPSCs, miniature inhibitory postsynaptic currents; PRL, propofol.

To further investigate the effects of the four drugs on the neurons, the neurons were both individually exposed to PRL (20 mM), EFV (4 mM), AZT (4 mM), and 3TC (4 mM) for 1 h, respectively, and simultaneously exposed to these four drugs for 1 h. The patch clamp was employed to detect the AP of each group of neurons. We found that the AP amplitude in the PRL (20 mM) group and LPA‐1‐h groups was significantly decreased (*p* < 0.01, Figure 3A). As PRL inhibits neuronal activity by enhancing the function of GABAaRs, we detected the mIPSCs, which are a common indicator of the function of GABAaRs. The electrophysiological results showed that the mIPSC amplitude was significantly increased in the LPA‐1‐h group and PRL (20 mM) group (*p* < 0.01, Figure 3B). However, no significant differences were observed between the other groups.

To exclude the effect of PRL on GABAaRs, we added Bicuculline (10 μM), a GABAaR antagonist,^33^ to the LPA‐1‐h group and PRL group. In the PRL (20 mM) + Bicuculline group, the mIPSC amplitude was decreased (*p* < 0.01, Figure 3B), while the AP amplitude was increased (*p* < 0.01, Figure 3A). Nevertheless, the AP amplitude was still significantly decreased in the LPA‐1‐h + Bicuculline group (*p* < 0.01, Figure 3A), even though the mIPSC amplitude was significantly decreased (*p* < 0.01, Figure 3B). In addition, the glucose concentration of the LPA‐1‐h + Bicuculline group did not differ significantly from that of the LPA‐1‐h group but was significantly lower than that of the Ctrl group (*p* < 0.01, Figure 3C). Conversely, there was no significant change in the neuronal glucose concentration in the PRL (20 mM) group or PRL (20 mM) + Bicuculline group (Figure 3C).

### 3.3 Glut3 expression

**FIGURE 4:**
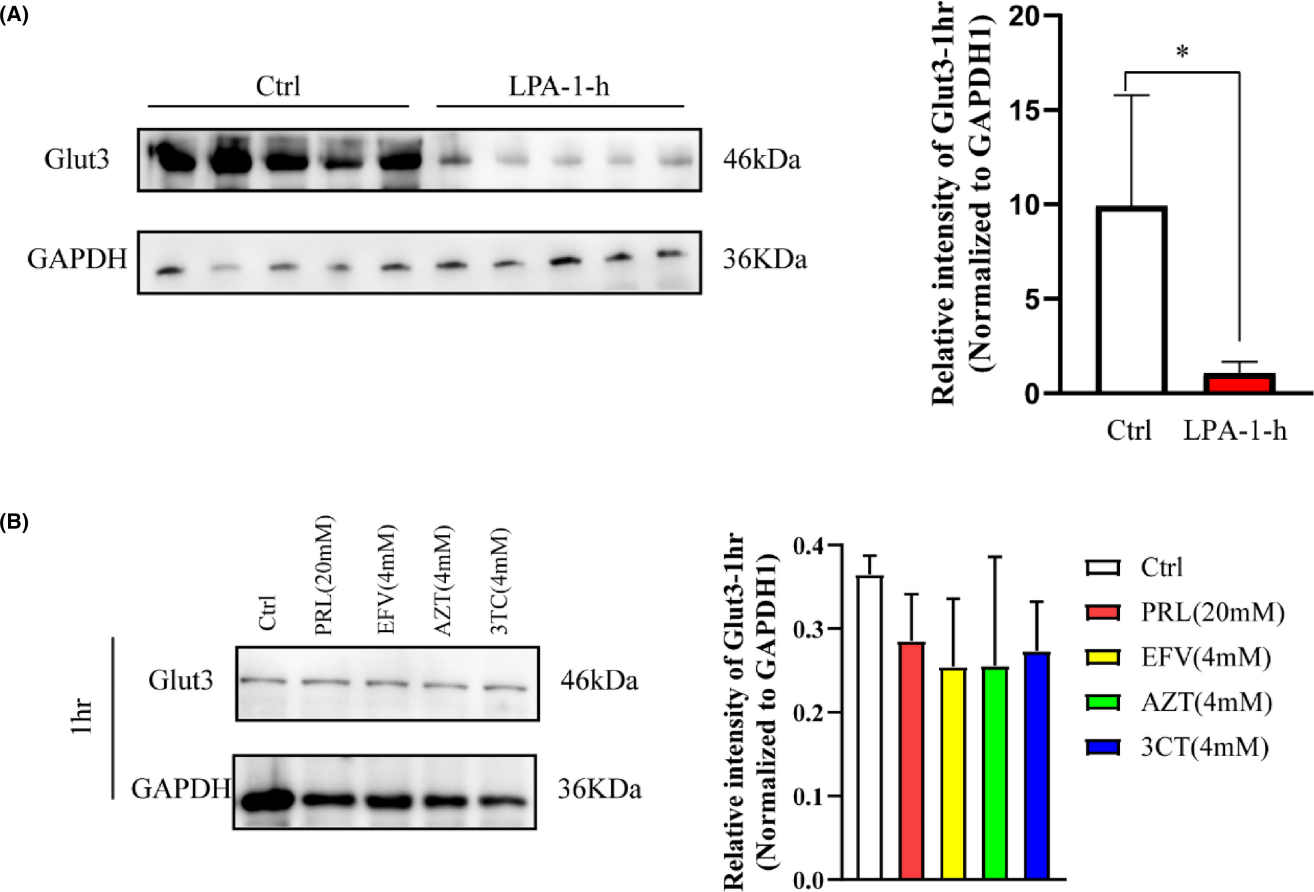
The expression of Glut3 (Western blot). (A) Effects of PRL and the ART drugs on Glut3 expression: The expression of Glut3 was decreased after neuron exposure to the 5 drugs for 1 h. (B) The effects of each individual drug on Glut3 expression: the expression of Glut3 did not change significantly. *n* = 5, *p < 0.05, versus Ctrl. 3TC, lamivudine; ART, antiretroviral therapy; AZT, zidovudine; Ctrl, control; EFV, efavirenz; Glut3, glucose transporter 3; PRL, propofol.

**FIGURE 5:**
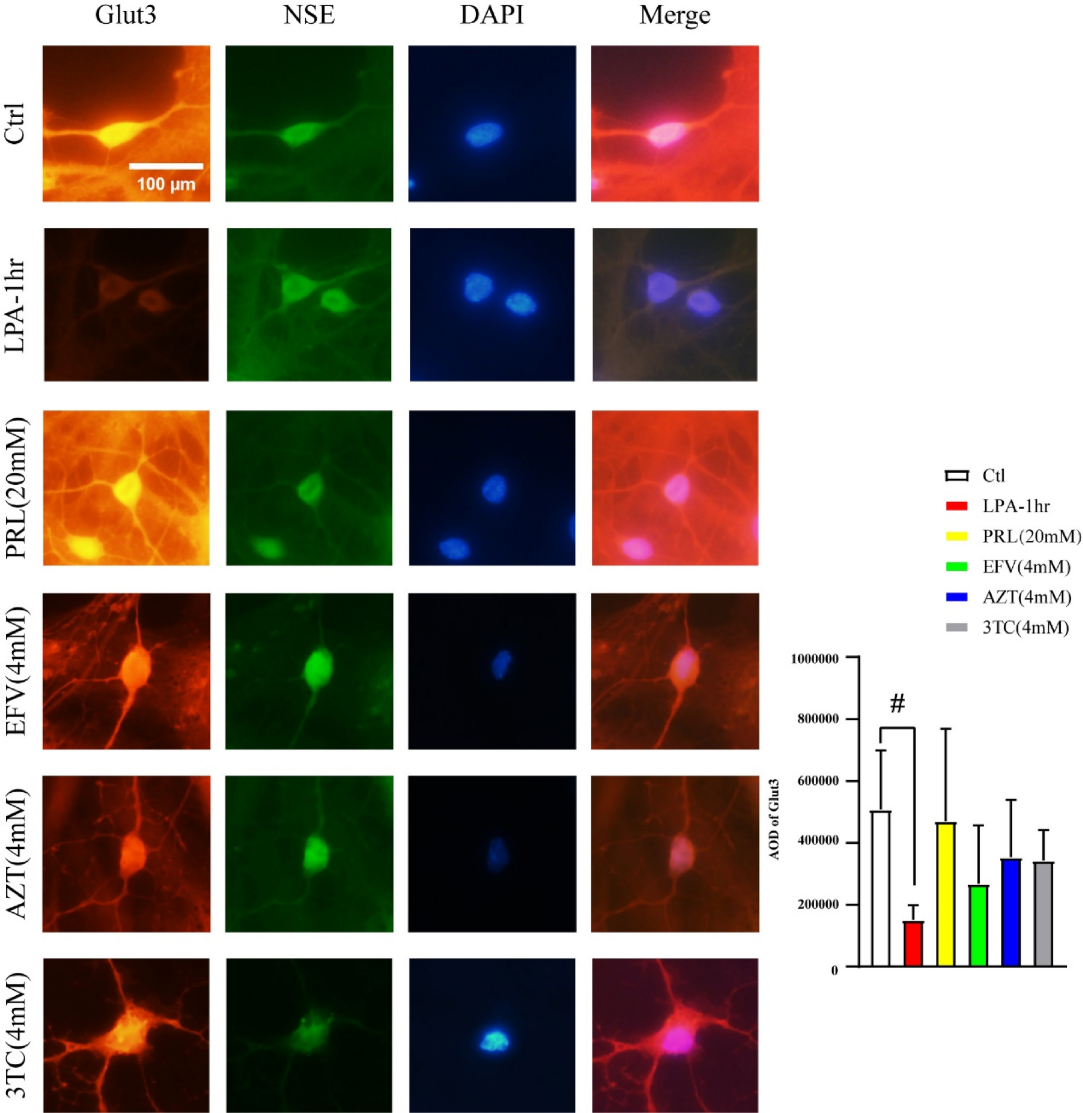
The AOD of Glut3 (immunofluorescence, ×600). The AOD of the Glut3 group was decreased in the LPA‐1‐h group, but no significant changes were observed in the other groups. Scale: 100 μm. *n* = 5, ^#^ *p* < 0.01. 3TC, lamivudine; AOD, average optical density; AZT, zidovudine; Ctrl, control; DAPI4′, 6‐diamidino‐2‐phenylindole; EFV, efavirenz; Glut3, glucose transporter 3; LPA, low PRL + ART concentration; NSE, neuron specific enolase; PRL, propofol.

The Western blot results showed that the expression of Glut3 was significantly decreased in the LPA‐1‐h group (*p* < 0.05, Figure 4A). Next, we investigated the expression of Glut3 in the neurons that had been individually exposed to PRL (20 mM), EFV (4 mM), AZT (4 mM), and 3TC (4 mM), respectively, for 1 h. The expression of Glut3 did not change significantly in the PRL (20 mM) group, EFV (4 mM) group, AZT (4 mM) group, and 3TC (4 mM) group (Figure 4B). The IF results indicated that the AOD of Glut3 in the neurons was significantly decreased in the LPA‐1‐h group (*p* < 0.01, Figure 4), but was not significantly different in the other groups (Figure 5).

## 4 DISCUSSION

HIV is a neurotropic virus that is highly sensitive to the CNS.^34^ Both PRL and ART drugs have a certain degree of neurotoxicity, which may cause secondary damage to the CNS.^10^, ^14^ Thus, close attention should be paid to protecting the CNS of HIV patients who receive PRL and ART simultaneously. To better protect the CNS, the potential mechanisms by which the drugs damage the neurons must first be clarified. Suzuki et al.^35^ suggested that neuronal energy metabolism disorder was related to cell damage, and the correction of this disorder could protect neurons. The main energy source of the brain is glucose, which is an energy source and precursor for the biosynthesis of neurotransmitters.^36^, ^37^ However, it is not yet known whether there is a time sequence for energy metabolism disorder and cell damage.

We found that PRL combined with ART drugs reduced the survival rate of neurons, which was positively correlated with the drug concentration and exposure time. Further, we found that neuronal damage was an adverse reaction induced by the DDIs between PRL and the ART drugs. Low levels of glucose can cause irreversible damage to the CNS.^38^ We also found that the longer the neurons had low glucose levels, the lower the survival rate of the neurons. There was no significant damage to the neurons exposed to low concentrations of PRL and ART drugs for 1 h, but the glucose levels did begin to decrease. Moreover, as the concentration and exposure time of the drugs increased, the glucose level and survival rate of the neurons decreased. We demonstrated that the disturbance of neuronal energy metabolism preceded neuronal damage, and the longer the neurons had a low glucose level, the more severe the neuronal damage.

To further investigate the effect of energy metabolism disorder on neuronal activity, we detected the neuronal AP, which is the basic indicator of neuronal activity.^39^, ^40^ The neuronal AP amplitudes were significantly decreased, which suggests that these drugs inhibit neuronal activity. To determine whether the decreased AP amplitude was caused by the DDIs between PRL and the ART drugs or an individual drug, the neurons were exposed to each drug separately for 1 h. The electrophysiological results showed that the neuronal AP amplitudes were only decreased in the PRL (20 mM) group. However, PRL has been shown to inhibit neuronal activity by activating GABAaRs, which mediate mIPSCs.^15^ To determine whether the reduction of neuronal action potential was only affected by PRL, a GABAaR blocker, Bicuculline, was employed to antagonize the neural inhibitory effects. The mIPSC and AP amplitudes were increased in the neurons exposed to PRL. However, the AP amplitude was still significantly decreased in the LPA‐1‐h + Bicuculline group, even though the mIPSC amplitude was significantly decreased. This suggests that the decrease in AP amplitude is not merely a result of neural Inhibition by PRL.

We also examined the glucose levels of the neurons that were exposed to each drug. The glucose concentration results showed that the neurons exposed separately to each drug did not display decreased glucose levels. However, the neurons exposed to both PRL and ART drugs showed a significant decrease in glucose, which was not blocked by Bicuculline. These results suggest that the mechanism of neuronal inhibitory did not play a key role in the decreased neuronal activity. Thus, the DDIs between PRL and ART drugs may result in a low level of neuronal glucose metabolism.

The AP is related to Na^+^/K^+^‐ATPase (NKA),^41^ which transports Na^+^ and K^+^ by hydrolyzing ATP.^42^, ^43^ Glucose is also an important metabolic substrate for ATP. Combining PRL with ART drugs reduced the glucose content of the neurons, resulting in insufficient ATP production and inhibiting the AP of the neurons. Thus, we showed that the DDIs between PRL and ART drugs inhibited neuronal activity by affecting glucose metabolism and not by enhancing neuronal inhibition.

Glucose is an important product of the tricarboxylic acid cycle in neurons,^22^, ^23^ which is closely related to the biosynthesis of the excitatory neurotransmitter glutamate and the inhibitory neurotransmitter GABA.^44^, ^45^, ^46^, ^47^ In the process of obtaining energy, neurons consume glucose primarily through the Glut3 on the cell membrane. The concentration of glucose in the brain is significantly lower than that in the peripheral blood, but the Glut3 can transport glucose regardless of the concentration gradient.^48^ To verify whether the DDIs between PRL and ART drugs affected Glut3, we detected the expression of Glut3. The western blot and IF results indicated that the expression of Glut3 was significantly decreased in the neurons exposed to PRL and ART drugs. However, the expression of Glut3 was not significantly changed in the neurons that were exposed to each drug individually. Thus, the decreased expression of Glut3 may be related to the DDIs between PRL and ART drugs.

## 5 CONCLUSIONS

We found that PRL combined with ART drugs led to decreased glucose metabolism and cell death in neurons. More importantly, we found that decreases in glucose metabolism predated neuronal damage. It may be that these drugs interfere with the expression of Glut3 and thus reduce glucose levels in neurons. Notably, our study revealed that changes in Glut3 and glucose appear earlier than cell damage, and thus may serve as indicators of CNS dysfunction. Thus, close attention should be paid to the following two points when using PRL and ART drugs in HIV‐infected patients: (I) the glucose level in the cerebrospinal fluid, which may be an indicator of CNS injury and (II) energy metabolism disorder, which should be treated to avoid structural damage to the CNS. Owing to the limitations of the study, We did not conduct further analysis in vivo.

## AUTHOR CONTRIBUTIONS

*Conception and design*: Sijun Li and Yanqing Zheng. *Administrative support*: Fengyao Wu. *Provision of study materials or patients*: Qian Long. *Collection and assembly of data*: Honghua Shao. *Data analysis and interpretation*: Gang Liang. *Manuscript writing*: All authors. *Final approval of manuscript*: All authors.

## FUNDING INFORMATION

This work was supported by the Key Research and Development Plan of Guangxi Province of China (Gui‐Ke, No. AB19110012) and the Youth Natural Science Foundation of Guangxi Province of China (no. 2022GXNSFBA035509).

## CONFLICT OF INTEREST STATEMENT

The authors have no conflict of interest to declare.

## Data Availability

The data supporting the findings of this study are available from the corresponding author upon request.
